# MAGI-MS: multiple seed-centric module discovery

**DOI:** 10.1093/bioadv/vbac025

**Published:** 2022-04-29

**Authors:** Julie C Chow, Ryan Zhou, Fereydoun Hormozdiari

**Affiliations:** 1 Department of Biochemistry and Molecular Medicine, UC Davis Genome Center, University of California, Davis, Davis, CA 95616, USA; 2 Department of Computer Science and Engineering, University of California, Merced, Merced, CA 95343, USA; 3 Department of Biochemistry and Molecular Medicine, UC Davis MIND Institute, Sacramento, CA 95817, USA

## Abstract

**Summary:**

Complex disorders manifest by the interaction of multiple genetic and environmental factors. Through the construction of genetic modules that consist of highly coexpressed genes, it is possible to identify genes that participate in common biological pathways relevant to specific phenotypes. We have previously developed tools MAGI and MAGI-S for genetic module discovery by incorporating coexpression and protein interaction networks. Here, we introduce an extension to MAGI-S, denoted as Merging Affected Genes into Integrated Networks—Multiple Seeds (MAGI-MS), which permits the user to further specify a disease pathway of interest by selecting multiple seed genes likely to function in the same molecular mechanism. By providing MAGI-MS with seed genes involved in processes underlying certain classes of neurodevelopmental disorders, such as epilepsy, we demonstrate that MAGI-MS can reveal modules enriched in genes relevant to chemical synaptic transmission, glutamatergic synapse and other functions associated with the provided seed genes.

**Availability and implementation:**

MAGI-MS is free and available at https://github.com/jchow32/MAGI-MS.

**Supplementary information:**

Supplementary data are available at *Bioinformatics Advances* online.

## 1 Introduction

The extensive genetic and phenotypic heterogeneity characteristic of complex disorders indicates that the interaction of multiple genes underlies etiology (Parenti *et al.*, 2020). The development of protein–protein interaction (PPI) and coexpression networks has aided in the identification of networks of genes hypothesized to belong to the same functional module and contribute to specific pathways (Chen *et al.*, 2020; Parikshak *et al.*, 2015).

Previously, we described a method called MAGI-S used to dissect complex phenotypes, such as epilepsy, by producing modules seeded from a single gene associated with the phenotype of interest (Chow *et al.*, 2019). We demonstrated that independently providing MAGI-S single seed neurodevelopmental disorder (NDD) genes with varying degrees of association with epilepsy revealed modules enriched in (i) non-synonymous coding *de novo* variation in affected NDD cases relative to controls, (ii) genes associated with epilepsy and (iii) *de novo* mutation specifically retrieved from epilepsy cohorts, suggesting that MAGI-S can uncover networks of genes relevant to a complex disorder.

We introduce an extension to the existing method MAGI-S (Chow *et al.*, 2019), referred to as Merging Affected Genes into Integrated Networks—Multiple Seeds (MAGI-MS). MAGI-MS permits the user to select multiple seed genes from which to construct modules, using either the average or minimum coexpression of other genes relative to the selected seeds during gene score assignment. As a result, modules constructed by MAGI-MS are significantly enriched in specific disease pathways in which the provided seed gene(s) participate. In addition, we have normalized gene scoring prior to seed pathway generation such that seed pathways do not preferentially consist of genes that are generally highly expressed. Furthermore, we have simplified the process of running the compiled MAGI-MS program by providing example commands, sample input files and suggested parameter combinations for ease of use.

## 2 Methods

**Fig. 1.**
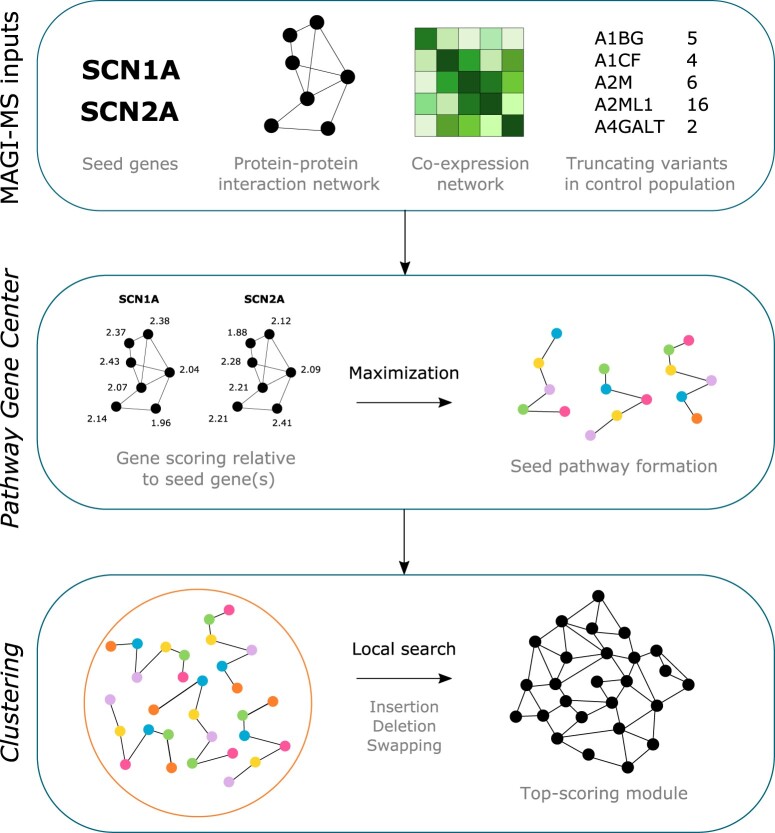
General methods overview of MAGI-MS. User-selected seed gene(s), a PPI network, a coexpression network and loss-of-function mutations observed in a control population are provided as input to construct modules specific to biological pathways associated with the provided seed genes. During *Pathway Gene Center*, scores are assigned to genes to describe their degree of coexpression with seed gene(s), and seed pathways consisting of high-scoring genes are formed. During *Clustering*, seed pathways are merged and refined to produce candidate modules

MAGI-MS uses a PPI network, coexpression network, deleterious mutations within a control population and seed gene(s) to create genetic modules that satisfy constraints related to PPI connectivity and degree of coexpression amongst module genes (Supplementary Data). In the following experiments, we use PPIs retrieved from the HPRD and the STRING databases (Keshava Prasad *et al.*, 2009; Szklarczyk *et al.*, 2011), RNA-seq data from the BrainSpan: Atlas of the Developing Human Brain as the coexpression network (Miller *et al.*, 2014) and control variants from the NHLBI Exome Sequencing Project (ESP; http://evs.gs.washington.edu/EVS/; Supplementary Data). Briefly, MAGI-MS assigns a score (Equation 1) to every gene within the PPI network (Fig. 1, Supplementary Data). High-scoring seed pathways are created by the use of a modified color-coding algorithm to find simple paths that maximize the summation of scores associated with genes (Hormozdiari *et al.*, 2015). Seed pathways are then merged into clusters by a random walk, and clusters are improved incrementally by local search to yield top-scoring modules.

To assess the ability of MAGI-MS to dissect a complex phenotype, we provided MAGI-MS with six pairs of seed genes, where each pair consists of genes observed to participate in a similar biological function (Szklarczyk *et al.*, 2021; *CHD8-CREBBP*, *CHD8-CTNNB1*, *GABRA3-GABRB1*, *GRIN2A-GRIN2B*, *SCN1A-SCN2A* and *SHANK2-SHANK3*). We additionally provided MAGI-MS with seed genes that are not hypothesized to participate in the same pathways (*SCN1A-CTNNB1* and *GRIN2A-GRIN2B-ADNP*), randomly selected gene pairs (*BCAS2-SHC1*, *RPL22L1-GEMIN2* and *RPL39L-LRRK2*) and up to 20 genes in the same pathway (long-term potentiation; Supplementary Data). To confirm the presence of relevant functional enrichment and cell-type-specific expression, modules were provided to the tools Enrichr and Cell-type-Specific Expression Analysis (CSEA) and respective enrichment scores were compared (Kuleshov *et al.*, 2016; Xu *et al.*, 2014); we also compared the functional enrichment of MAGI-MS modules with clusters containing seed genes that were generated via PPI clustering algorithms, including MCODE and CytoCluster applications within Cytoscape (version 3.9.0; Bader and Hogue, 2003; Li *et al.*, 2017; Shannon *et al.*, 2003; Supplementary Data).

The number of seeds needed to achieve maximum enrichment may vary depending on the degree of connectivity amongst seed genes and other genes, the extent of shared genes among other related biological pathways and the number of genes in the targeted pathway. It is possible to systematically prioritize candidate seeds by first providing *Pathway Gene Center* with initial seed gene(s) either arbitrarily or based on prior knowledge of importance. *Pathway Gene Center* scores every gene in the PPI network and returns these scores prior to seed pathway construction, where the highest scoring gene displays the highest degree of connectivity with the previously supplied seed(s). Thus, given a list of genes of interest in the same pathway and by retrieving their gene scores, the user can effectively rank candidate seeds and identify a set of seeds to maximize relevant enrichment. A script to prioritize candidate seeds is provided at https://github.com/jchow32/MAGI-MS.

## 3 Results

Given pairs of seed genes involved in the same biological pathway, MAGI-MS produces modules that have significant overlap with modules seeded from either seed gene alone (Supplementary Tables S1 and S2). On average, 49.5% and 61.4% of the genes in paired modules exist, using either minimum or average coexpression values during gene score assignment, respectively, in either of the singly seeded modules. Modules generated by MAGI-MS (Supplementary Table S1) generally display significantly larger enrichment scores (referred to as ‘combined scores’) compared to singly seeded modules produced by MAGI-S (Supplementary Table S2). For example, most paired-seed modules display significantly greater combined scores or odds ratios in enriched Kyoto Encyclopedia of Genes and Genomes (KEGG) pathways or Gene Ontology (GO) Biological Processes than at least one of the modules produced by a single constituent seed gene.

If MAGI-MS is successively supplied with multiple seeds known to participate in the same pathway, increasingly large enrichment scores can be observed up to a certain point, after which additional seeds do not yield increased enrichment in the targeted pathway. Supplementary Table S3 compares the combined scores of up to 20 seeds involved in the long-term potentiation KEGG pathway. Even while excluding seed genes from the module during functional enrichment analysis, constructed modules using three to five seeds genes yield increased enrichment in the long-term potentiation pathway compared to using fewer seeds.

Compared to PPI clustering algorithms such as MCODE and CytoCluster, MAGI-MS produces modules that are seeded from user-selected genes and are specific to pathways in which seed gene(s) participate, whereas modules derived from PPI clustering methods may not necessarily contain a user’s specific seed genes of interest. For PPI clusters containing any seed gene supplied to MAGI-MS, direct comparison of enrichment terms indicate that MAGI-MS shows significantly greater enrichment scores for KEGG pathways and GO Biological Processes compared to MCODE clusters (Supplementary Table S4). Additional modules generated using recent PPI and coexpression data are supplied in Supplementary Table S5.

Modules with paired seeds related to the epilepsy phenotype (*GABRA3-GABRB1*, *GRIN2A-GRIN2B* and *SCN1A-SCN2A*) were enriched in terms such as long-term potentiation, chemical synaptic transmission, among others and showed selective expression in deep cortical neurons (Supplementary Table S1). For seed gene pairs related to more general NDD and autism phenotypes (*CHD8-CREBBP* and *CHD8-CTNNB1*), we observe an enrichment in chromatin organization and regulation of transcription. For modules constructed with seed genes that do not participate in the same biological function (*SCN1A-CTNNB1*, *BCAS2-SHC1*, *RPL22L1-GEMIN2* and *RPL39L-LRRK2*), a module was not formed due to low-scoring seed pathways. For a combination of seeds that do not all participate in the same pathway (*GRIN2A-GRIN2B-ADNP*), a module is produced due to the sufficient degree of connectivity between seeds in the same pathway; however, decreased enrichment in relevant pathways is observed. For example, compared to the *ADNP* (26.76) or *GRIN2A-GRIN2B* (19.61) module, the overall score of the *GRIN2A-GRIN2B-ADNP* module is reduced to 17.65, and functional enrichment of pathways specific to *GRIN2A-GRIN2B*, such as long-term potentiation and glutamatergic synapse, is reduced or absent (Supplementary Table S1). Pathways previously significantly enriched in the *ADNP* module are also reduced, such as ubiquitin-mediated proteolysis, the transforming growth factor-beta signaling pathway and the Wnt signaling pathway. The choice of multiple seed genes from pathways with similar biological function is critical to form a module that is useful for the dissection of a specific phenotype.

## 4 Conclusion

We present an extension to the existing method MAGI-S, denoted as MAGI-MS, which improves upon MAGI-S by (i) permitting the discovery of genetic modules that are specific to certain biological functions by selection of multiple seed genes involved in a pathway of interest, (ii) normalizing gene score assignment to reduce bias during seed pathway formation and (iii) yielding comparable or increased functional enrichment in relevant biological pathways. MAGI-MS is freely available with updated user guides for parameter and input choices.

## Funding

This work has been supported partly by the National Science Foundation (NSF) [award DBI-2042518] to F.H.

*Conflict of Interest*: none declared.

## Supplementary Material

vbac025_Supplementary_Data
