# Supplementary material for: MAGI-MS: multiple seed-centric module discovery
Source: Bioinform Adv. 2022 Apr 29;2(1):vbac025. doi: 10.1093/bioadv/vbac025 (PMC9710684; doi:10.1093/bioadv/vbac025)
Supplement: vbac025_Supplementary_Data [file vbac025_supplementary_data.zip › SupplementaryData.pdf]

Supplementary Data

MAGI-MS takes as input a protein-protein interaction (PPI) network, a co-expression network, loss-of-function mutation from control populations, and multiple user-selected seed gene(s). For direct comparison to modules generated from MAGI-S, the PPI network was retrieved from HPRD and STRING (Keshava Prasad *et al.*, 2009; Szklarczyk *et al.*, 2011), using interactions with confidence scores greater than 700 and experimental scores greater than 400. Normalized RPKM values were retrieved from the BrainSpan: Atlas of the Developing Human Brain (Miller *et al.*, 2014) (V6). Truncated variants from the NHLBI Exome Sequencing Project (ESP) (http://evs.gs.washington.edu/EVS/) were used. More recent PPIs from the STRING database (version 11.5) and co-expression data (BrainSpan: Atlas of the Developing Human Brain V10) were used to construct modules displayed in **Supplementary Table 5**.

Genes within modules constructed by MAGI-MS must satisfy constraints related to 1) degree of connectivity in the PPI network, 2) high pairwise co-expression among module genes, and 3) restriction of the number of deleterious loss-of-function mutations in module genes from a control population as described in the Supplementary Material of MAGI (Hormozdiari *et al.*, 2015).

Pathway Gene Center

To construct modules, first, a score is assigned to every gene within the PPI network denoting its degree of co-expression with the seed gene(s). This score ($G_{s,i}$) (**Equation 1**), as in MAGI-S (Chow *et al.*, 2019) and MAGI-MS, is calculated as follows:

$$G_{s,i} = ((H_1)(H_2)) / N^2 \qquad \textbf{Equation 1}$$

For every gene to be scored (s) relative to a seed gene (i), the score ($G_{s,i}$) is the product of two values which describe the ranking of co-expression between (s) and (i), referred to as 'coexpression(s, i)', relative to all other genes in the PPI network. H_1 is the number of pairwise comparisons for which the [co-expression(s, i) > co-expression(i, another gene in the PPI network)]. H_2 is the number of pairwise comparisons for which the [co-expression(s, i) > co-expression(s, another gene in the PPI network)]. N is the total number of genes within the PPI network.

Compared to MAGI-S, MAGI-MS differs in that 1) gene scores are calculated for every gene relative to each seed gene rather than a single seed gene, and 2) gene scores are normalized. For example, if two seed genes are provided, then any particular gene will have two scores, where each score is associated with a different seed gene. For each seed gene (i), individual gene scores are z-scored (**Equation 2**), such that the scores for any particular seed gene possess a mean score of 0 with standard deviation of 1.

$$z\_score = (G_{s,i} - \mu_i) / \sigma_i \qquad \textbf{Equation 2}$$

Following z-scoring, a final score for every gene in the PPI network is assigned by either taking the average (-avg) or minimum (-min) score among candidate scores from each seed. A larger score indicates a greater degree of co-expression with the seed genes. By calculating an average score, final gene scores will reflect the average degree of co-expression that the gene possesses with seed genes. By using a minimum score, final gene scores will reflect the largest degree of co-expression observed with any of the seed genes *i* to *j* that were provided.

After final scores have been assigned to every gene in the PPI network, seed pathways are formed to ensure that modules consist of genes that display a high degree of connectivity. Seed pathways consist of *h* genes, where MAGI-MS seeks to maximize the summation of gene scores within the seed pathway by randomly coloring genes with *h* different colors and finding the colorful path via dynamic programming. The use of a modified color coding algorithm permits MAGI-MS to limit the number of deleterious mutations observed from a control population while finding the colorful path (Hormozdiari *et al.*, 2015; Alon *et al.*, 1995). By simultaneously maximizing the summation of gene scores in seed pathways and limiting the number of deleterious mutations observed in a control population, MAGI-MS thus identifies non-random sets of interacting genes. During *Pathway Gene Center*, a total of 16,000 seed pathways are generated using 1,000 iterations of combinations of number of loss-of-function mutations allowed in the control population (0, 1, 2, 3) and number of genes within seed pathways (h = (5, 6, 7, 8)), written to 16 files (*BestPaths* files).

Clustering

During the clustering process, seed pathways generated from *Pathway Gene Center* are merged into high scoring clusters via a random walk. To improve candidate modules, a local search is performed in which individual genes are removed, added, or swapped and the module score is returned. Modules that both satisfy the mentioned constraints and result in an increased module score following local search are produced. The user may independently run several iterations of *Clustering* with varied parameters after a single completed execution of *Pathway Gene Center* to compare multiple candidate modules.

Parameter selection

Parameters may be modified during the *Clustering* phase. The minimum (-l) and maximum (-u) size of the constructed module can be specified. We recommend varying the minimum average co-expression of the module (-avgCoExpr, recommended range: 0.425-0.52) and the minimum PPI density of the modules (-avgDensity, recommended range: 0.085-0.14). For seeds with generally low pairwise co-expression values, -avgCoExpr can be further reduced. The parameter (-i) is simply an integer used for the initialization of a random number generator. In practice, the number of deleterious loss of function mutations allowed in genes in the module (-m = 6), the minimum ratio of seed scores allowed (-a = 0.5), and minimum pairwise co-expression value allowed (-minCoExpr = 0.01) are not varied.

Time complexity

MAGI-MS has two main steps: pathway construction and clustering. The pathway construction runs in $O(2^k)$, where k is the maximum length of pathways generated. We bound k to be O(log h), where h is the size of the input graph. The clustering step is linear to the number of pathways constructed (n). Thus, clustering runtime is O(n). On average, for two seed genes, *Pathway Gene Center* completes in 5.30 hours and *Clustering* completes in 1.97 hours running on Ubuntu 16.04.7 LTS via an AMD Opteron(tm) Processor 6380 (Architecture: x86-64, CPUs: 64, CPU MHz: 1396.406).

Enrichment analyses among MAGI-MS, MAGI-S, and PPI clustering methods

The Cell-type Specific Expression Analysis (CSEA), Specific Expression Analysis (SEA), and Tissue Specific Expression Analysis (TSEA), and Enrichr tools (Xu *et al.*, 2014; Kuleshov *et*

al., 2016) were applied to each of the 6 modules constructed with pairs of seed genes (CHD8-CREBBP, CHD8-CTNNB1, GABRA3-GABRB1, GRIN2A-GRIN2B, SCN1A-SCN2A, and SHANK2-SHANK3). Enriched KEGG, Gene Ontology (GO) Biological Process, and Online Mendelian Inheritance in Man (OMIM) Expanded terms are displayed for each of the 6 modules in **Supplementary Table 1.** The GRIN2A-GRIN2B-ADNP and ADNP modules are also displayed in the GRIN2A-GRIN2B-ADNP tab of **Supplementary Table 1** with corresponding enrichment terms. Modules constructed using single seeds via MAGI-S are shown in **Supplementary Table 2**. In the 'summary' tab of **Supplementary Table 2**, associated p-values resulting from paired t-tests comparing combined scores, odds ratios, and adjusted p-values among shared enrichment terms between a paired seed gene module and corresponding singly-seeded modules are shown. Directional (1-sided) paired t-tests test the hypotheses that 1) the combined score is greater for the paired seed modules than the singly-seeded module, 2) the odds ratio is greater for the paired modules versus the singly-seeded, and 3) the adjusted p-value is smaller for the paired modules versus the singly-seeded. Non-directional (2-sided) t-tests test the hypothesis of equality in shared enrichment terms. In **Supplementary Table 4**, the 'summary' tab similarly indicates significance of directional and non-directional paired t-tests of paired seed modules with clusters resulting from the MCODE and CytoCluster (HC-PIN) PPI clustering methods within the Cytoscape program (version 3.9.0) (Shannon *et al.*, 2003; Bader and Hogue, 2003; Li *et al.*, 2017). Default parameters were used during PPI clustering (MCODE: degree cutoff=2, haircut=enabled, node score cutoff=0.2, k-core=2, max. depth=100; CytoCluster HC-PIN: weak=enabled, threshold=2.0, complexSize threshold=3). The enrichment scores of a paired module and a PPI cluster were compared if the PPI cluster contained at least one seed gene existing within paired seeds, which include CHD8, CREBBP, CTNNB1, GABRA3, GABRB1, GRIN2A, GRIN2B, SCN1A, SCN2A, and SHANK3. If significant selective expression was detected via CSEA, SEA, and or TSEA for the provided module, respective selective expression plots are displayed in **Supplementary Figure 1**.

To further compare the enrichment of a specific pathway given multiple seeds that participate in the targeted pathway, up to 20 seeds (**Supplementary Table 3**) in the long-term potentiation KEGG pathway were provided to MAGI-MS. To determine the sequence by which a seed was selected and appended to the list of seeds used as inputs to *Pathway Gene Center*, the following procedure was used. After the user selects one or more seed genes involved in the targeted pathway, additional seeds are prioritized from a list of candidate seeds (all remaining genes in the targeted pathway, or a user-selected list) by calculating the gene scores of candidate seeds as per **Equation 1**. The candidate seed with the largest gene score is then selected as the next seed to append to the previous list of seeds. To select another seed, gene scores are again calculated using the newly appended list of seeds. For example, after providing the seeds GRIN2B-GRIN2A to MAGI-MS, PRKCA possessed the largest gene score among genes in the long-term potentiation pathway and was thus appended as a seed gene (GRIN2B-GRIN2A-PRKCA). Given the seeds GRIN2B-GRIN2A-PRKCA during seed pathway creation, GRIA2 was then identified as the next highest scoring candidate seed. A total of 20 modules seeded via repeated prioritization of genes in the long-term potentiation pathway from the initial seeds GRIN2B-GRIN2A are displayed in **Supplementary Table 3** with associated long-term potentiation pathway enrichment scores.

A) CHD8-CREBBP (-min). SEA: slight enrichment in early mid-fetal cortical tissue.

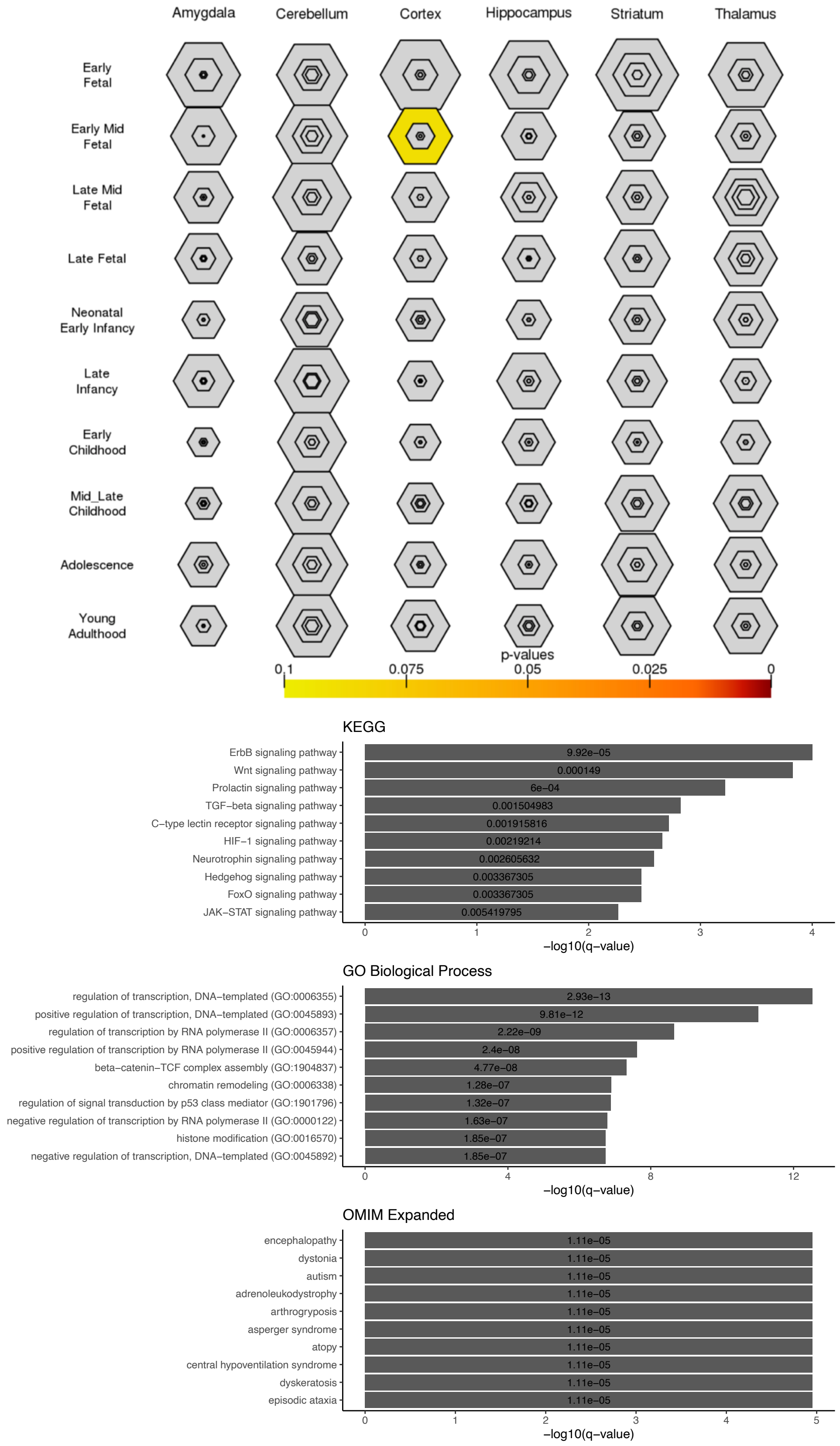

B) CHD8-CREBBP (-avg). SEA: slight enrichment in early mid-fetal cortical tissue. CSEA: enrichment in rods (retina).

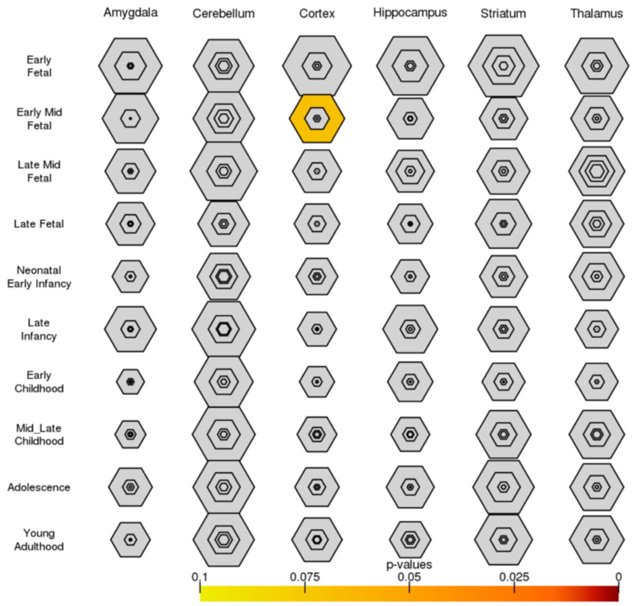

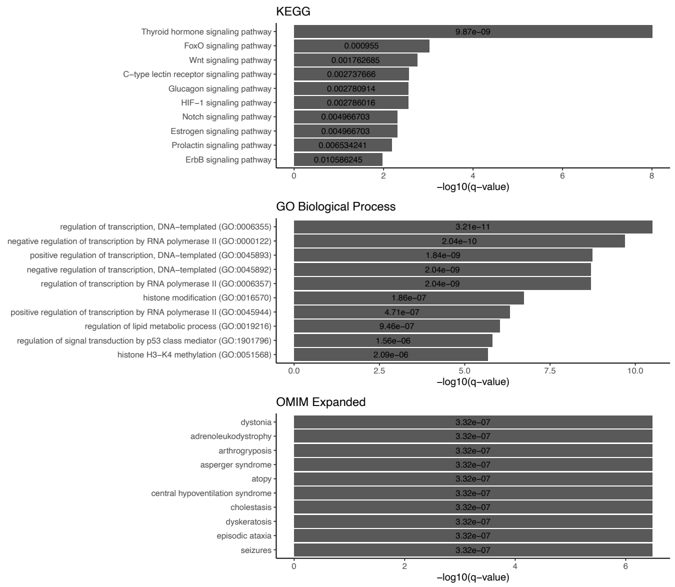

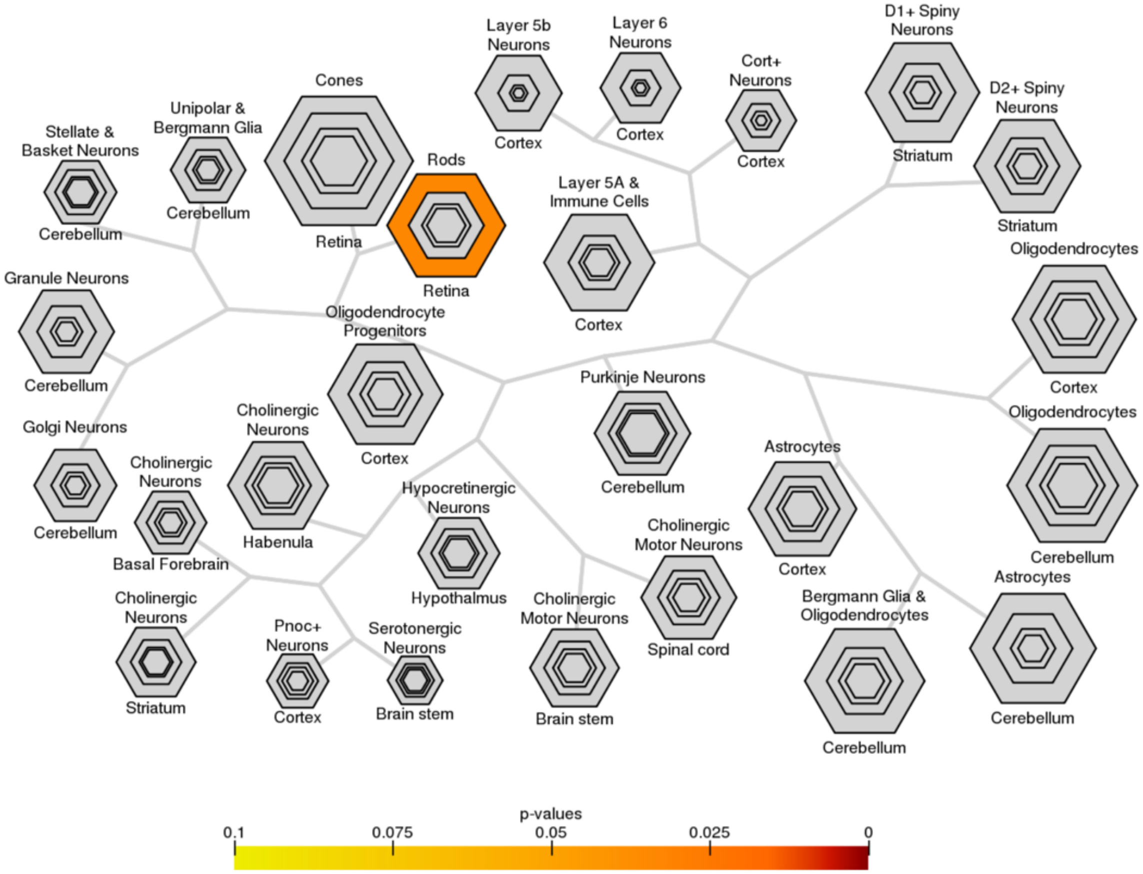

C) CHD8-CTNNB1 (-min). SEA: slight enrichment in early fetal striatum.

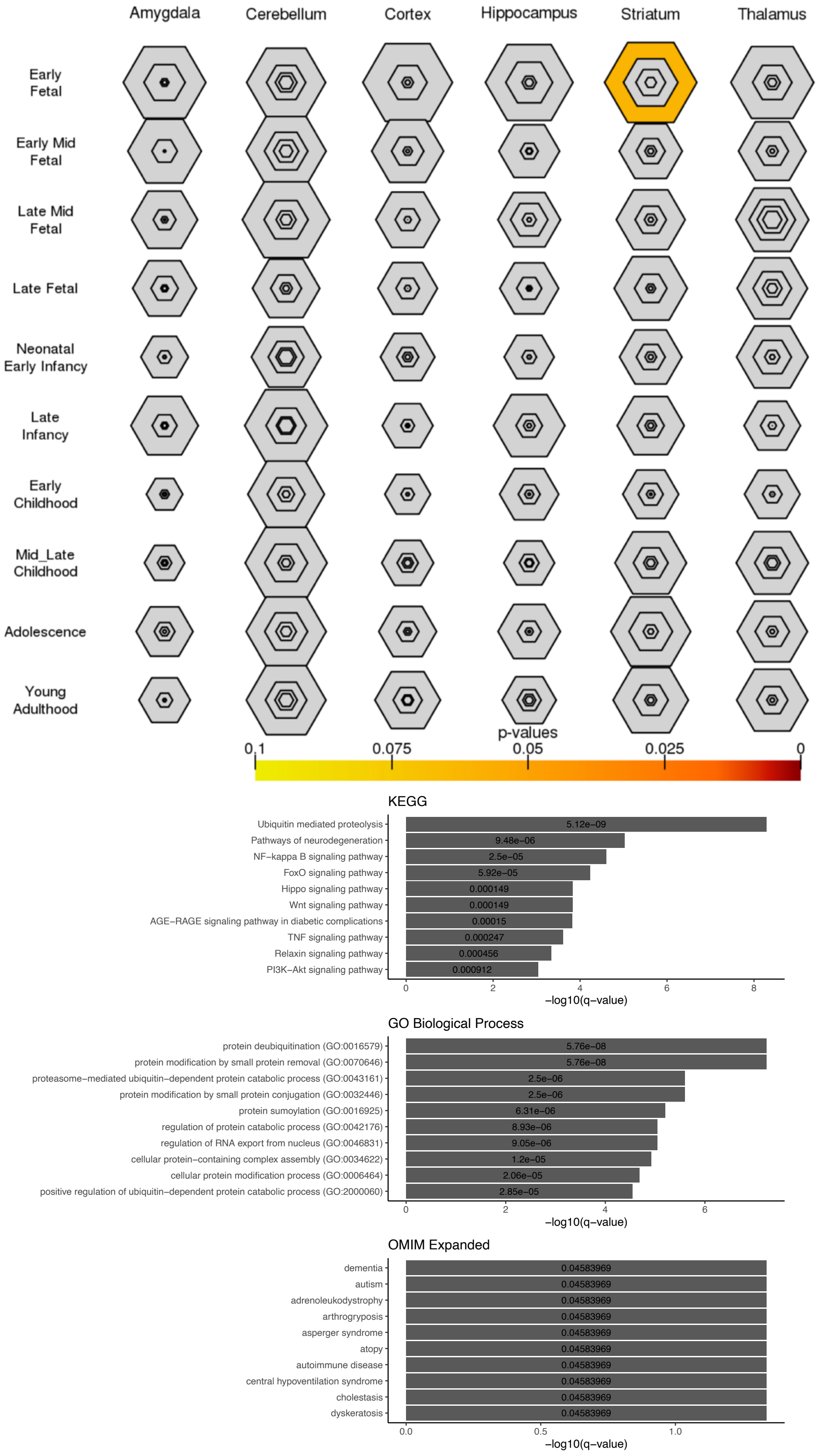

D) CHD8-CTNNB1 (-avg). No significant enrichment via CSEA, SEA, or TSEA tools.

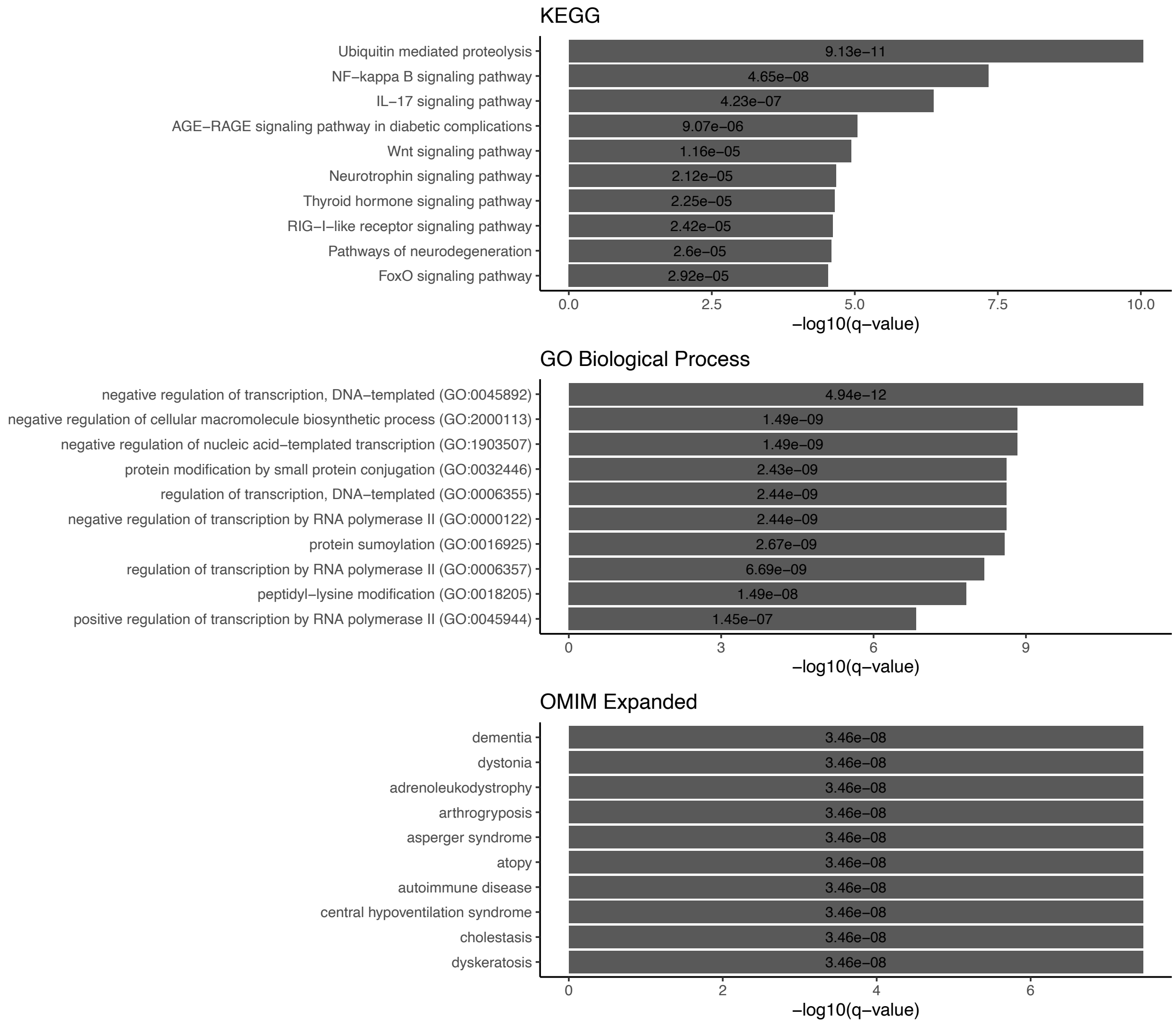

E) GABRA3-GABRB1 (-min). SEA: Enrichment in cortical tissue during young adulthood.

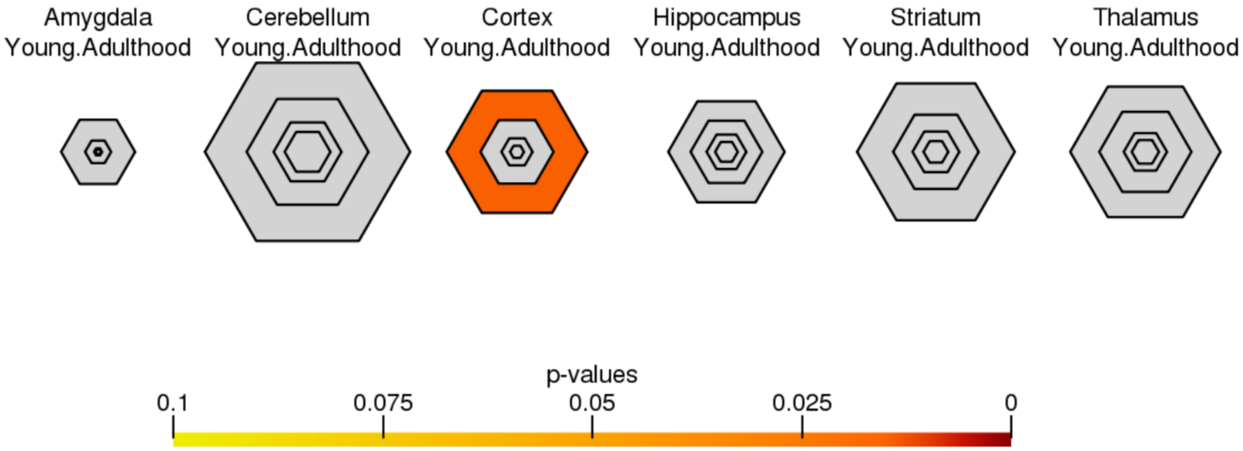

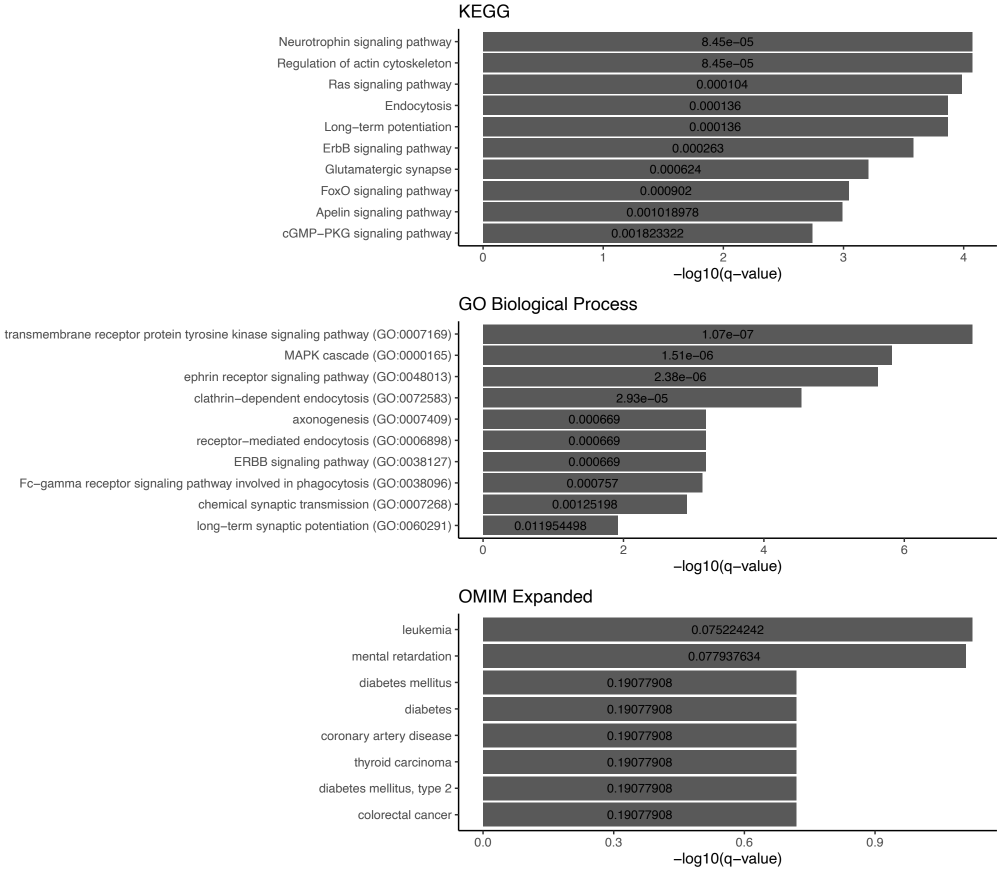

F) GABRA3-GABRB1 (-avg). TSEA: enrichment in brain. SEA: enrichment in cortical tissue during young adulthood.

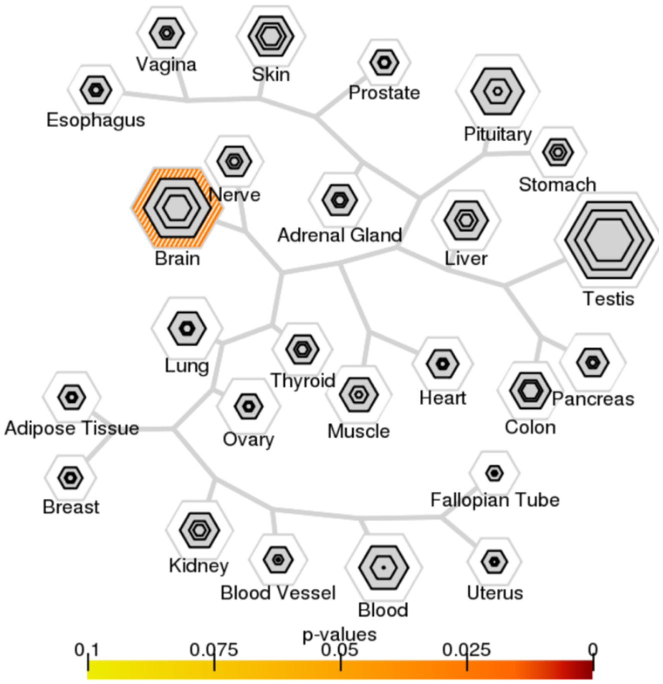

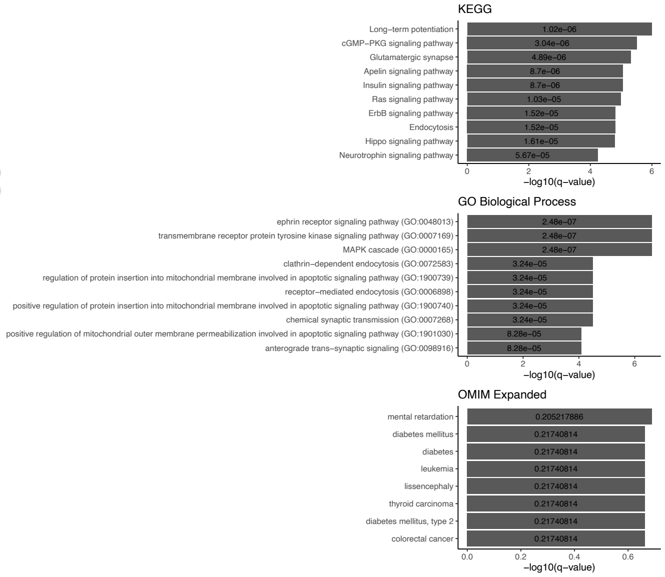

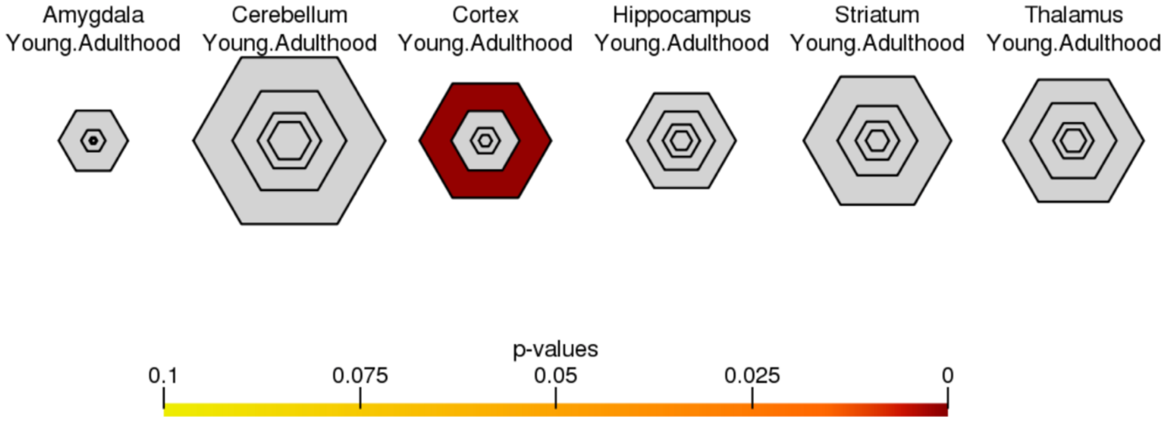

G) GRIN2A-GRIN2B (-min). CSEA: enrichment in layer 5b cortical neurons and D1+ spiny striatal neurons. SEA: increased enrichment in cortical tissues during young adulthood and neonatal early infancy. TSEA: increased enrichment in brain and pituitary gland.

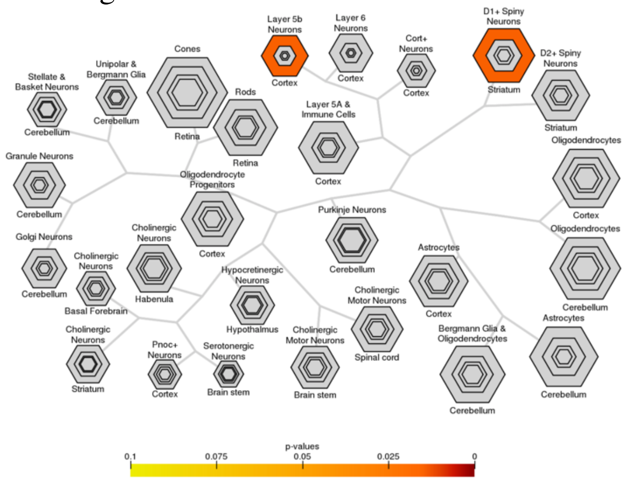

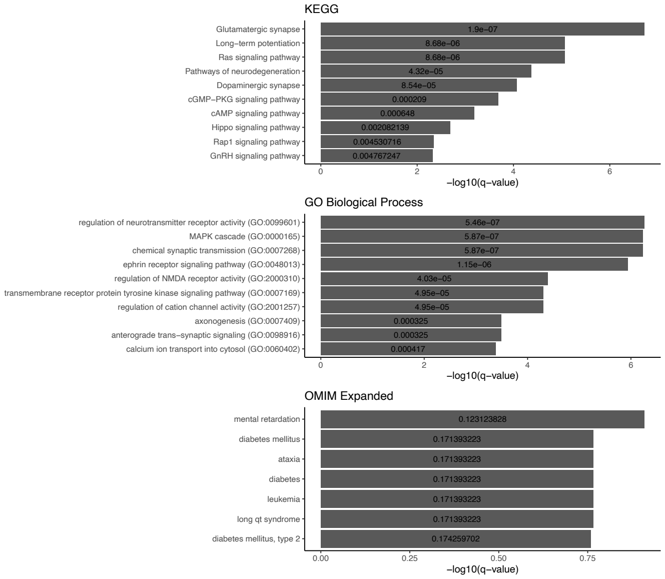

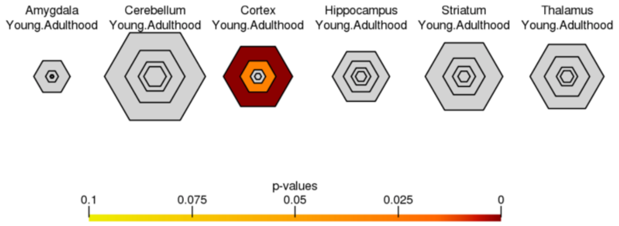

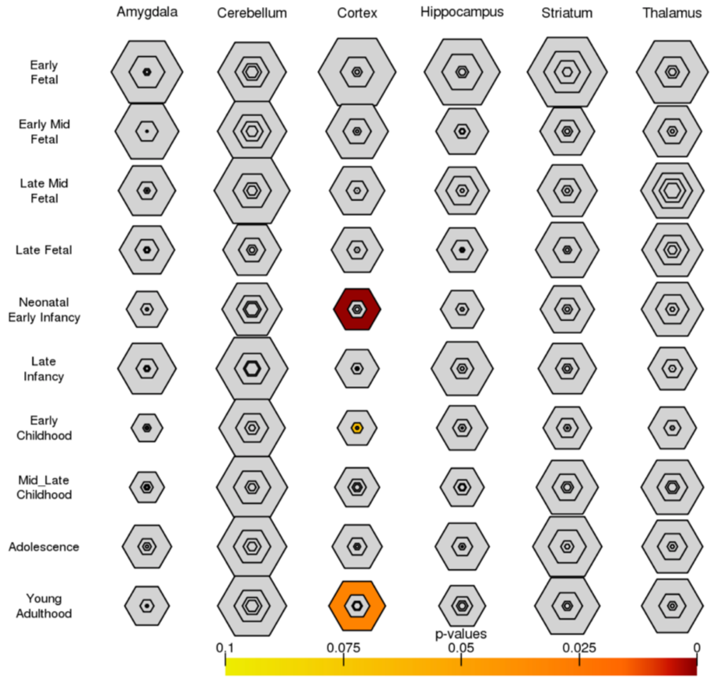

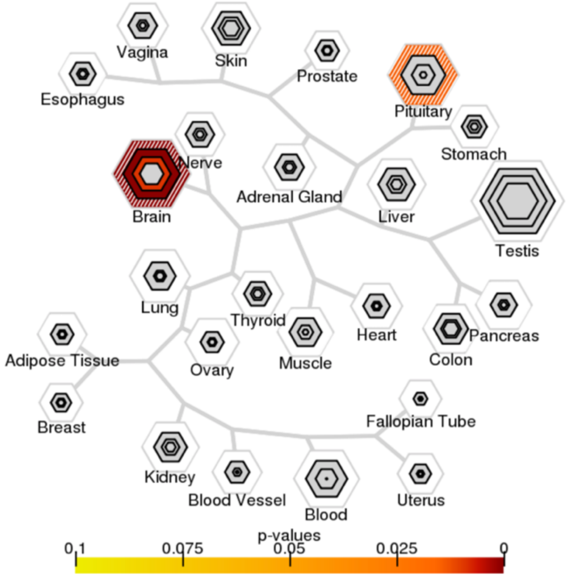

H) GRIN2A-GRIN2B (-avg). CSEA: enrichment in deep cortical neurons and D1+ spiny striatal neurons. SEA: increased enrichment in cortical tissues during neonatal early infancy and from early childhood to young adulthood. Enrichment in striatal tissue during early childhood. TSEA: increased enrichment in brain and pituitary gland.

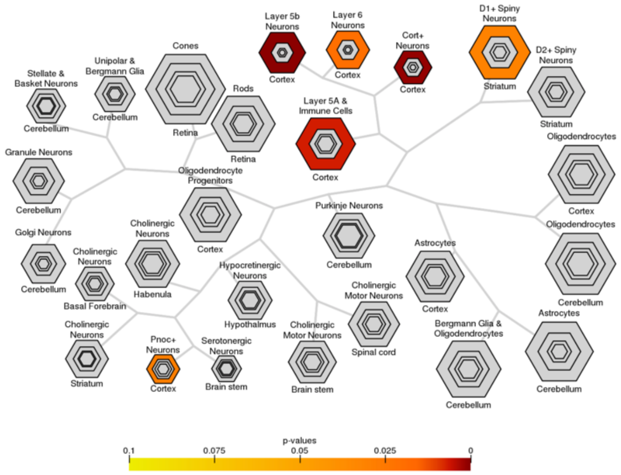

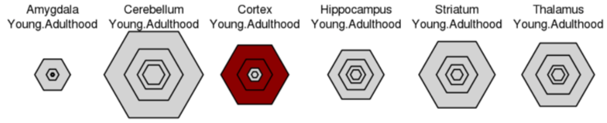

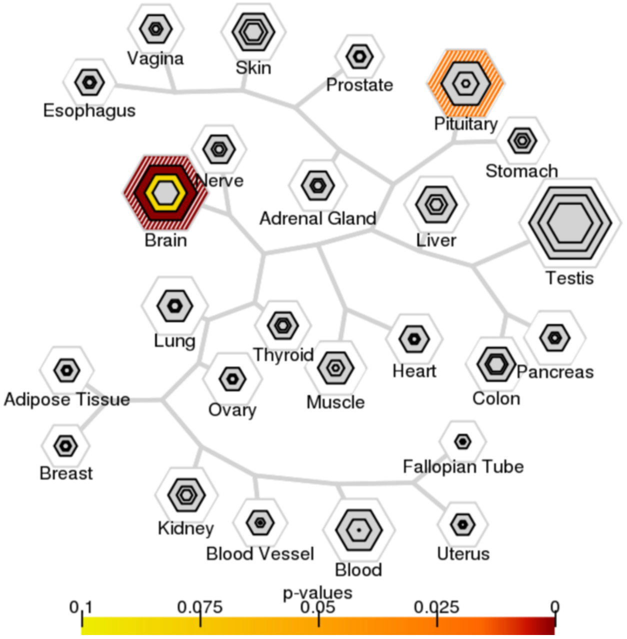

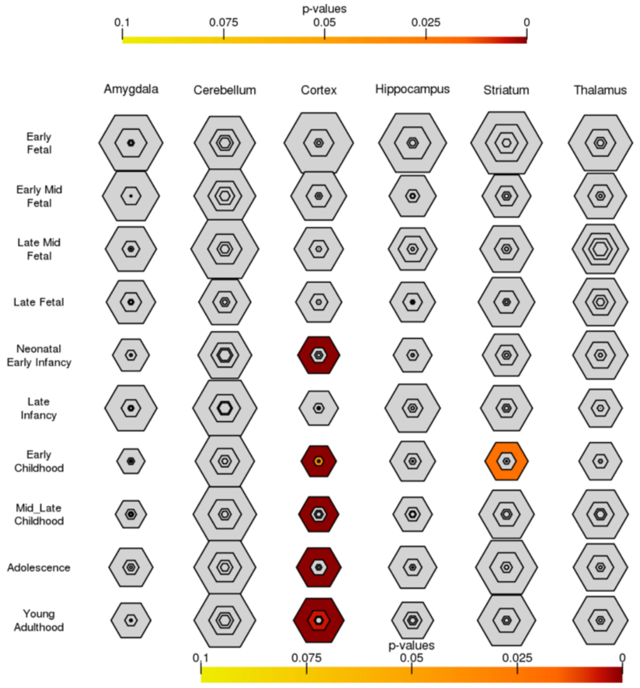

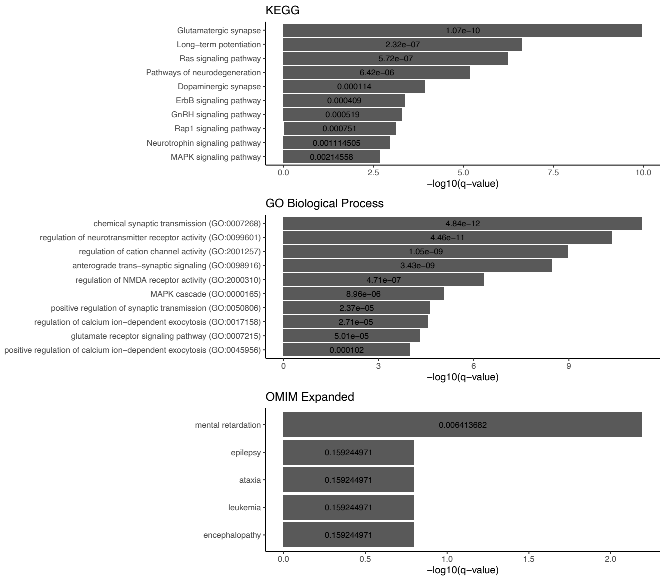

I) SCN1A-SCN2A (-min). CSEA: enrichment in layer 5b and 6 cortical neurons and D1+ spiny striatal neurons. SEA: widespread enrichment in the amygdala, cerebellum, cortex, hippocampus, and thalamus from neonatal early infancy to young adulthood. TSEA: increased enrichment in brain and pituitary gland.

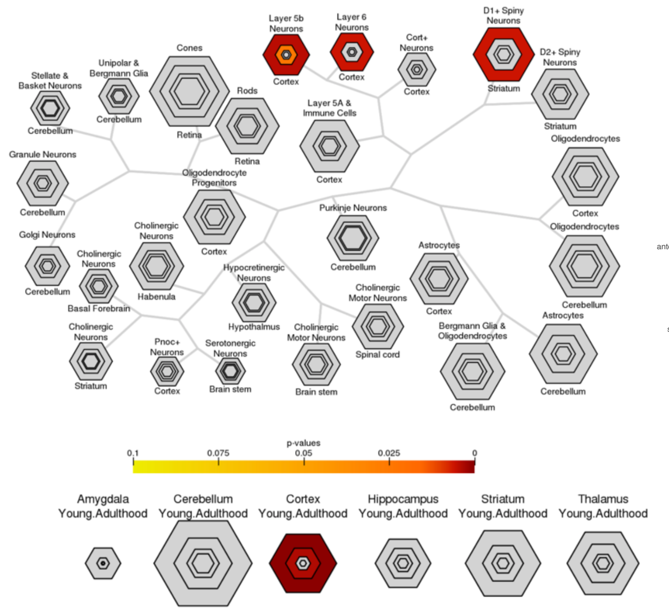

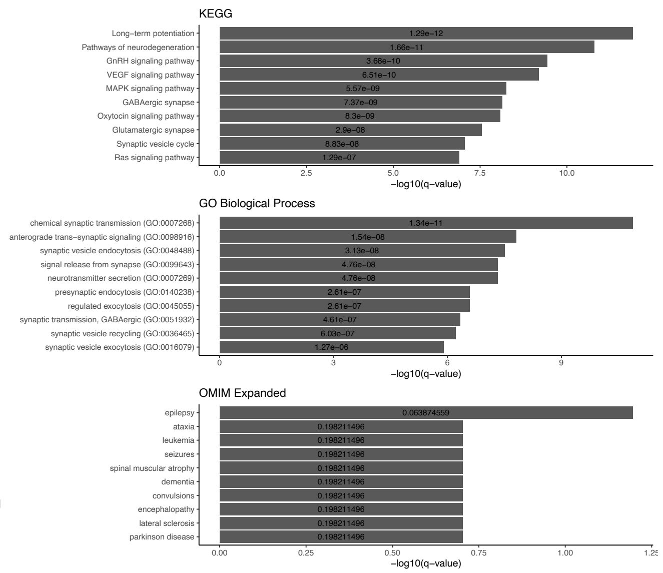

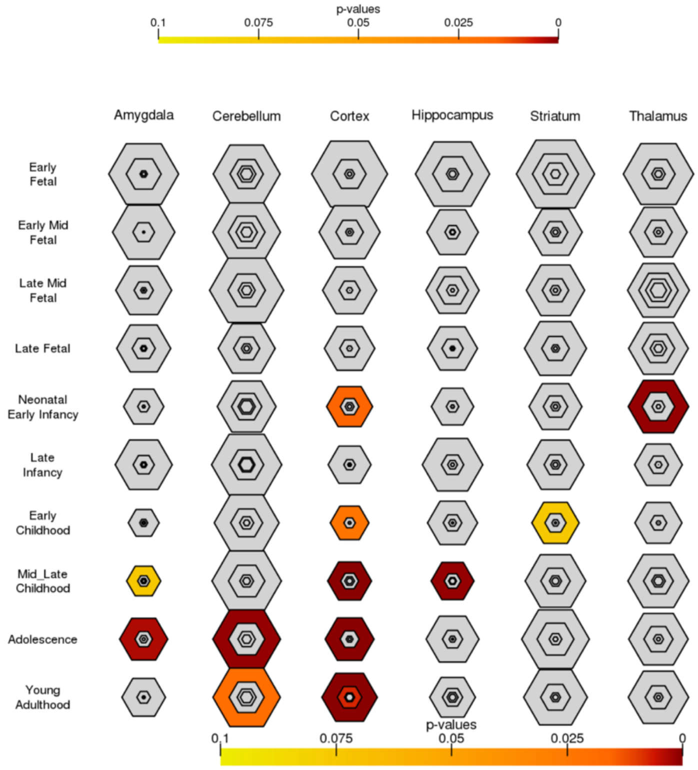

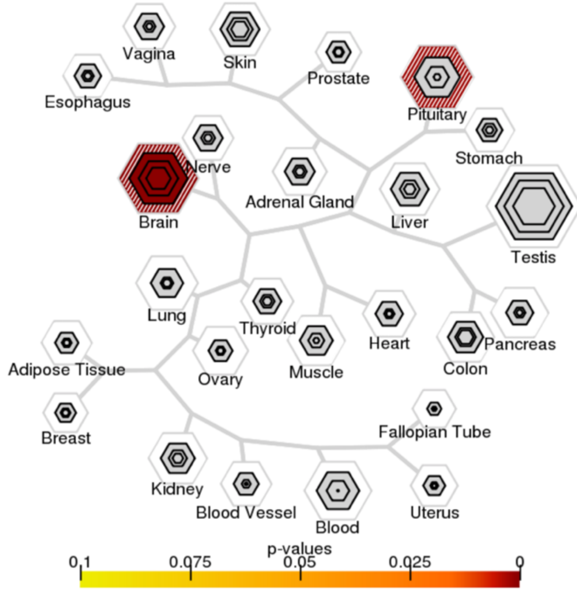

J) SCN1A-SCN2A (-avg). SEA: increased enrichment in cortical tissue during young adulthood, and widespread enrichment in the amygdala, cerebellum, cortex, from mid-late childhood to young adulthood. TSEA: increased enrichment in brain.

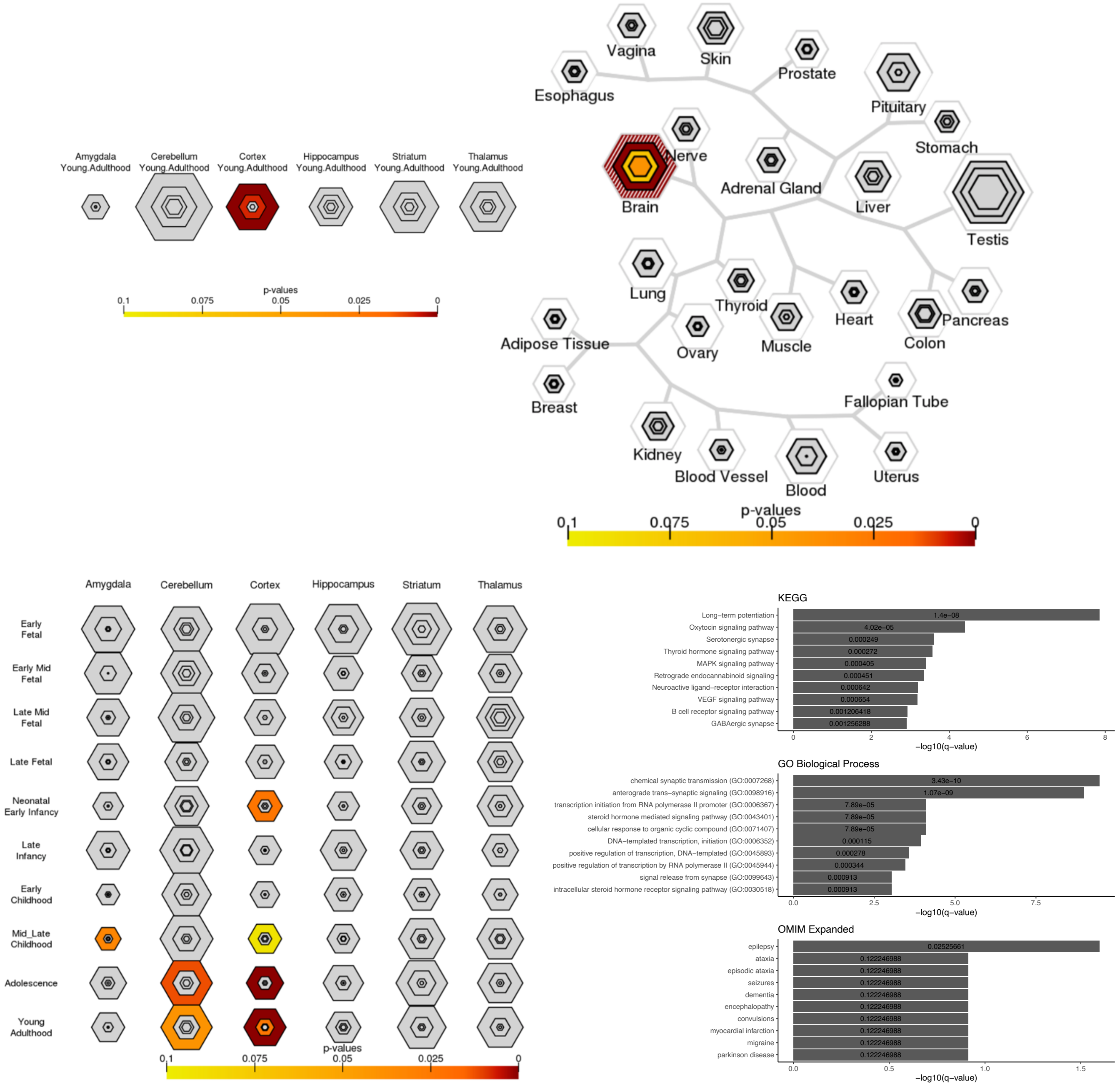

K) SHANK2-SHANK3 (-min). CSEA: enrichment in layer 5b, 5a, and 6 cortical neurons. SEA: enrichment in the amygdala, cortex, and thalamus from neonatal early infancy to young adulthood. TSEA: increased enrichment in brain and pituitary gland.

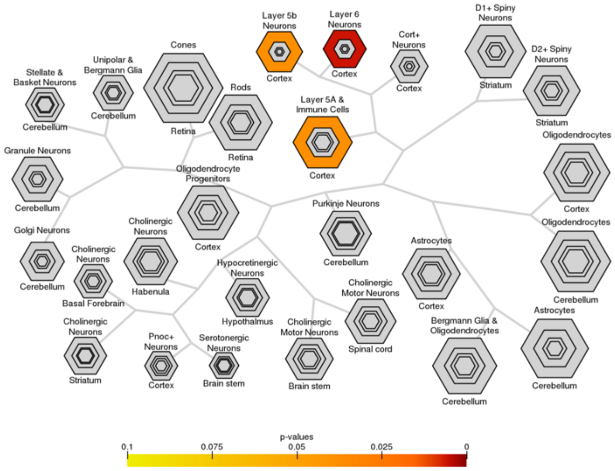

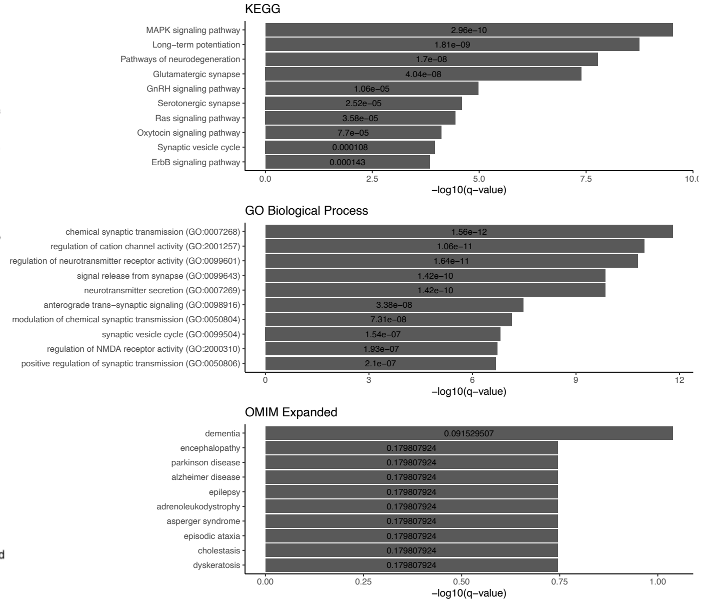

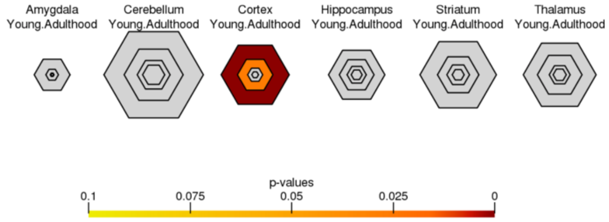

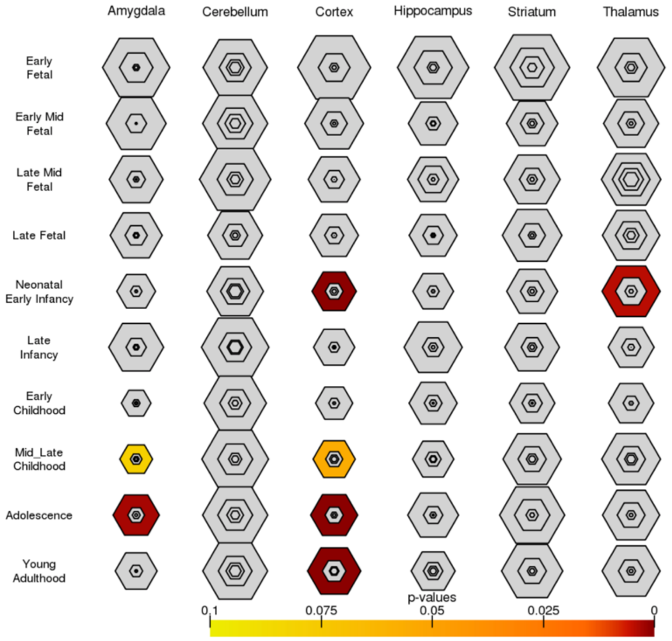

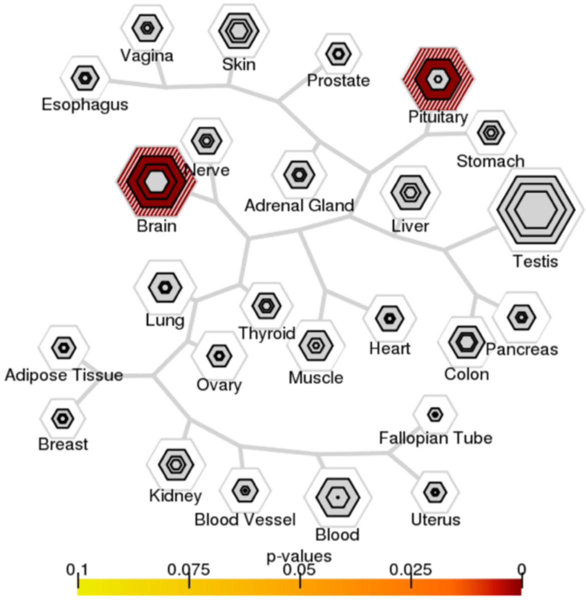

L) SHANK2-SHANK3 (-avg). CSEA: enrichment in layer 5b, 5a, and 6 cortical neurons and D1+ and D2+ spiny striatal neurons. SEA: enrichment in the amygdala, cortex, hippocampus, and thalamus from neonatal early infancy and early childhood to young adulthood. TSEA: increased enrichment in brain and pituitary gland.

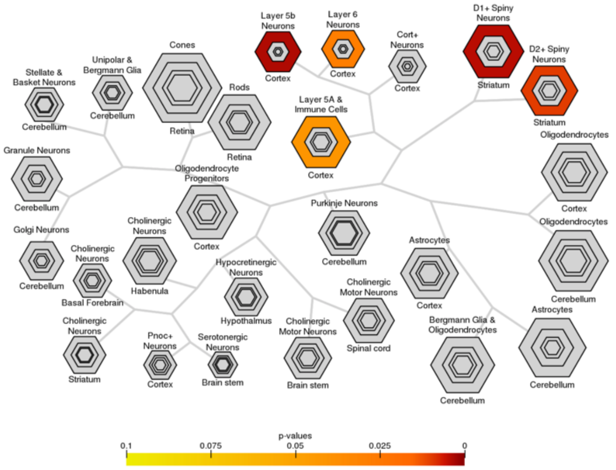

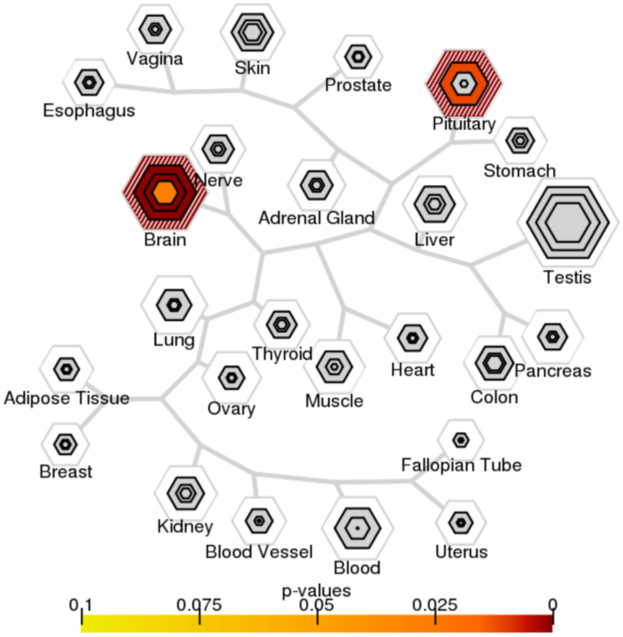

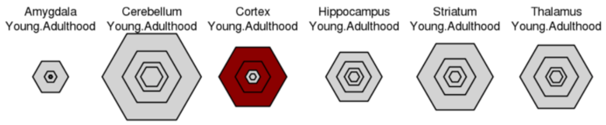

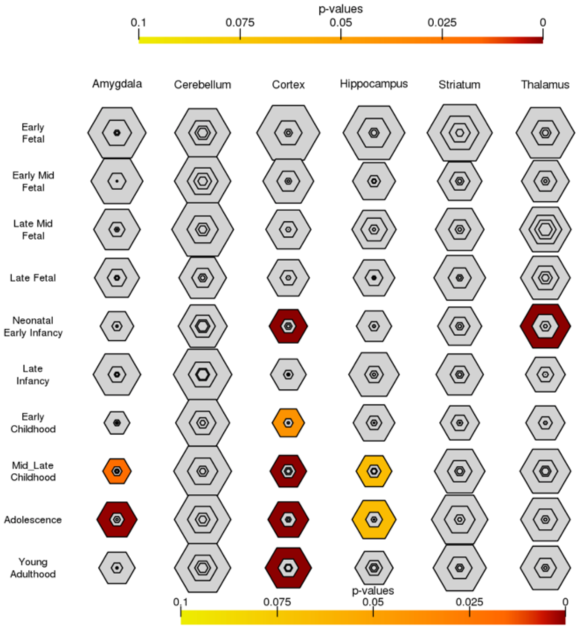

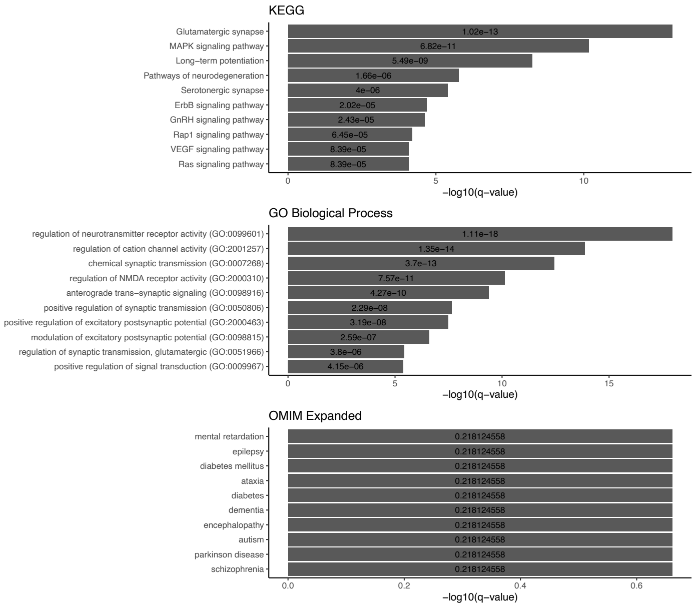

Supplementary Figure 1. Significant cell-type and tissue specific expression analysis for MAGI-MS modules. If significant selective expression is detected via the CSEA, SEA, and TSEA tools for a module generated using either average (-avg) or minimum (-min) co-expression values during score assignment in *Pathway Gene Center*, corresponding figures are shown (-min, -avg): A-B) CHD8-CREBBP, C-D) CHD8-CTNNB1, E-F) GABRA3-GABRB1, G-H) GRIN2A-GRIN2B, I-J) SCN1A-SCN2A, K-L) SHANK2-SHANK3. CSEA and SEA describe the significant enrichment of provided genes across cell-types in the human brain at various developmental time periods. TSEA identifies over-representation of provided (disease) genes with enriched expression in certain human tissues. Significance (p-value) in CSEA, SEA, and TSEA plots is indicated by color intensity, where red indicates p-values close to 0. Top KEGG, GO Biological Processes, and OMIM Expanded enrichment terms are displayed.

Supplementary Table 1. Modules produced via MAGI-MS for selected sets of seed genes. The summary tab displays the number of genes within each paired seed gene module, compared to the number of genes that are shared with respective singly-seed modules created by MAGI-S, using either average (-avg) or minimum (-min) co-expression during gene score assignment. Each paired seed gene tab displays genes within singly-seeded modules and paired modules generated using (-min) or (-avg) parameters, and associated KEGG, GO Biological Processes, and OMIM Expanded enrichment terms retrieved from Enrichr. The GRIN2A-GRIN2B-ADNP tab displays modules seeded using genes that participate in the same pathway (GRIN2A-GRIN2B, ADNP) and associated enrichment terms for the GRIN2A-GRIN2B-ADNP and ADNP modules.
Supplementary Table 2. Modules produced via MAGI-S. The summary tab displays the associated p-values of one-sided and two-sided paired t-test comparisons of Enrichr's Combined Score, odds ratio, and adjusted p-value for KEGG, GO Biological Processes, and OMIM Expanded enrichment terms that are shared between singly-seeded modules created by MAGI-S and paired seed gene modules created by MAGI-MS. Each tab corresponding to a single seed gene displays the associated module and its KEGG, GO Biological Processes, and OMIM Expanded enrichment terms.
Supplementary Table 3. Modules produced via MAGI-MS for up to 20 seeds in the long-term potentiation KEGG pathway. The summary tab displays the seeds in sequential order as they are appended to the list of seeds given to MAGI-MS according to their calculated gene score. Combined scores, odds ratios, and adjusted p-values for modules excluding or including seed genes are shown. Each tab label corresponds to the number of seeds used to generate a module, and each tab displays KEGG enrichment terms for the module while excluding or including seed genes.
Supplementary Table 4. Modules produced via MCODE and CytoCluster that contain seed genes. The summary tab displays associated p-values of one-sided and two-sided paired t-test comparisons of Enrichr's Combined Score, odds ratio, and adjusted p-value for KEGG, GO Biological Processes, and OMIM expanded enrichment terms that are shared between MCODE or CytoCluster clusters that contain seed genes and MAGI-MS modules.
Supplementary Table 5. Modules produced via MAGI-MS for selected pairs of seed genes using recent STRING (version 11.5) and the Atlas of the Developing Human Brain (version 10). The summary tab compares the number of genes, GO Biological Processes, and KEGG terms that are shared or unique among modules generated using older (STRING + HPRD + Allen Brain V6) or more recent (STRING 11.5 + Allen V10) inputs. In general, genes that are shared between modules generated using older and newer inputs constitute the majority of genes in modules generated using newer inputs. Each tab displays associated KEGG, GO Biological Processes, and OMIM Expanded enrichment terms retrieved from Enrichr for every pair of seed genes.

Alon,N. *et al.* (1995) Color-coding. *J. ACM*, **42**, 844–856.

Bader,G.D. and Hogue,C.W. (2003) An automated method for finding molecular complexes in large protein interaction networks. *BMC Bioinformatics*, **4**, 2.

Chow,J. *et al.* (2019) Dissecting the genetic basis of comorbid epilepsy phenotypes in neurodevelopmental disorders. *Genome Med.*, **11**, 65.

Hormozdiari,F. *et al.* (2015) The discovery of integrated gene networks for autism and related disorders. *Genome Res.*, **25**, 142–154.

Keshava Prasad,T.S. *et al.* (2009) Human Protein Reference Database—2009 update. *Nucleic Acids Res.*, **37**, D767–D772.

Kuleshov,M.V. *et al.* (2016) Enrichr: a comprehensive gene set enrichment analysis web server

2016 update. *Nucleic Acids Res.*, **44**, W90–W97.
Li,M. *et al.* (2017) CytoCluster: A Cytoscape Plugin for Cluster Analysis and Visualization of Biological Networks. *Int. J. Mol. Sci.*, **18**, 1880.
Miller,J.A. *et al.* (2014) Transcriptional landscape of the prenatal human brain. *Nature*, **508**, 199–206.
Shannon,P. *et al.* (2003) Cytoscape: a software environment for integrated models of biomolecular interaction networks. *Genome Res.*, **13**, 2498–2504.
Szklarczyk,D. *et al.* (2011) The STRING database in 2011: functional interaction networks of proteins, globally integrated and scored. *Nucleic Acids Res.*, **39**, D561–D568.
Xu,X. *et al.* (2014) Cell Type-Specific Expression Analysis to Identify Putative Cellular Mechanisms for Neurogenetic Disorders. *J. Neurosci.*, **34**, 1420–1431.
